# Paleopathology of a Lower Miocene Carettochelyid Turtle from the Moghra Formation, Egypt

**DOI:** 10.3390/biology15120980

**Published:** 2026-06-22

**Authors:** Andrea Guerrero, Adán Pérez-García, Mohamed K. AbdelGawad, Alberto Valenciano

**Affiliations:** 1 Grupo de Biología Evolutiva, Facultad de Ciencias, Universidad Nacional de Educación a Distancia (UNED), Avda. de Esparta s/n, 28232 Madrid, Spain; aguerrerobach@ccia.uned.es (A.G.); a.perez.garcia@ccia.uned.es (A.P.-G.); 2 Geology Department, Faculty of Science, Cairo University, Giza 12613, Egypt; 3 Departamento de Geodinámica, Estratigrafía, Paleontología, Faculty of Geological Sciences, Universidad Complutense de Madrid, 28040 Madrid, Spain; albvalen@ucm.es; 4 Research and Exhibitions Department, Iziko Museums of South Africa, Cape Town 8001, South Africa

**Keywords:** Testudines, Cryptodira, North Africa, paleopathology, osteomyelitis

## Abstract

This study examines two articulated peripheral plates of a carettochelyid turtle from the Moghra Formation (Early Miocene, 19.6–18.2 Ma) in Egypt. This research represents the first record of shell pathologies in an African carettochelyid. The specimens were analyzed through detailed macroscopic observation and computed tomography (CT) imaging. To characterize the observed anomalies, a comparative analysis was conducted based on standardized clinical and veterinary osteopathological methodologies. The results revealed significant external and internal structural alterations, including irregular globular outgrowths and internal remodeling evidenced by high-density bone formation. These features enable the identification of pathological conditions that differ from the bioerosive traces and other surface modifications that have been documented previously in the turtle fauna of the Moghra Formation.

## Simple Summary

We describe the first pathological shell modifications reported in an African carettochelyid turtle, based on two articulated shell plates from the Early Miocene Moghra Formation of Egypt. Macroscopic observations and computed tomography imaging revealed abnormal bone growth, necrotic areas, and extensive internal remodeling, consistent with an advanced infectious process. The degree of reactive bone formation indicates prolonged survival of the individual during disease progression. These findings provide new information on pathological processes in fossil turtles and on biological interactions in the aquatic Moghra paleoecosystem.

## 1. Introduction

**Figure 1:**
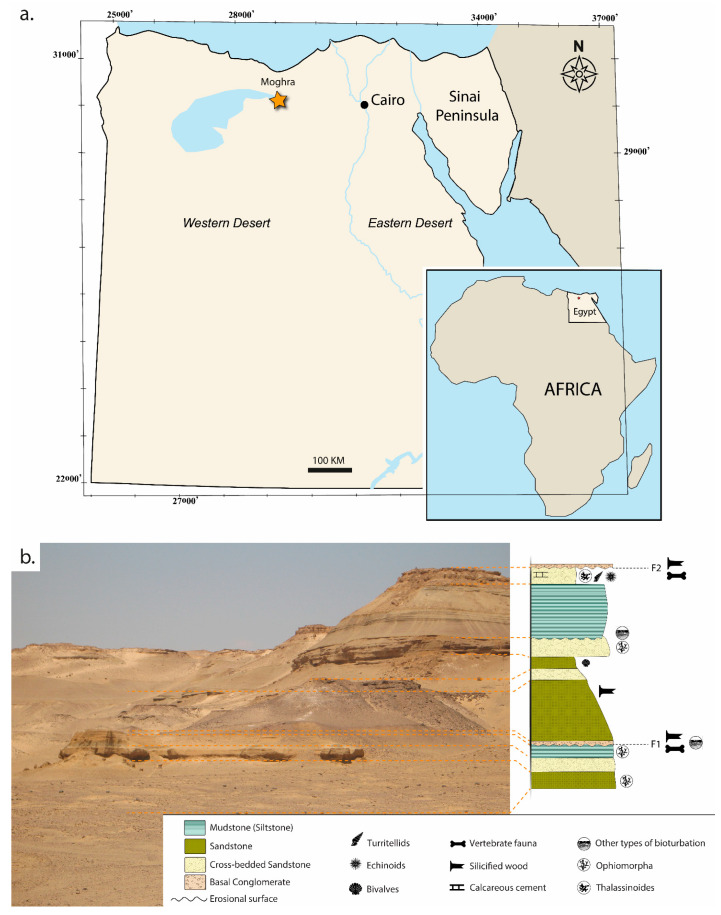
Geographic and stratigraphic context of the Moghra locality (Early Miocene, Egypt), where the studied specimens were recovered. (**a**) Geographic location of the Moghra locality, with inset showing the position of Egypt within Africa. (**b**) Stratigraphic section and corresponding field photograph of the lower units of the Moghra Formation. The schematic column illustrates the main lithostratigraphic units with estimated thicknesses, key vertebrate-bearing horizons (F1, F2), and the distribution of invertebrate fauna and trace fossils. This figure was extracted from [5,7] and subsequently modified by the authors.

The Moghra Formation (Early Miocene) (Figure 1) is known by a diverse vertebrate fauna represented by a mixture of marine, freshwater and terrestrial taxa, including mammals, osseous fishes, sharks, birds, crocodiles, squamates, and turtles [1,2,3,4,5,6,7,8,9,10,11,12,13,14]. This rich record has provided key insights into the palaeoecological dynamics and faunal composition of the North African Miocene.

Within this assemblage, turtle remains have proven particularly informative for reconstructing biotic interactions [15,16], as their shells frequently preserve surface modifications of biological and taphonomic origin. Previous studies have largely approached these features from an ichnological perspective. For instance, ref. [15] documented a variety of trace fossils, including pits and grooves on testudinid and podocnemidid shells, interpreting them as the result of invertebrate activity during the life of the host organisms [15]. However, comparable analyses have not yet been conducted on representatives of the Carettochelyidae in the Moghra Formation. This group is characterized by a distinctive shell morphology, including a reduced or absent keratinous covering and a naturally textured external bone surface, which may complicate the identification and interpretation of pathological or biogenic modifications.

**Figure 2:**
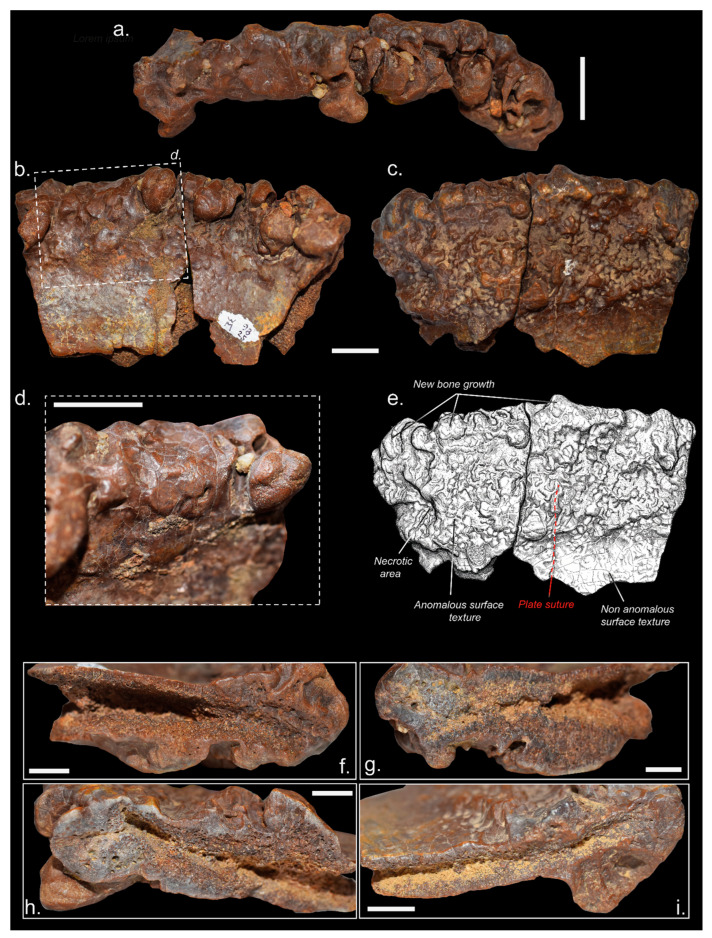
Two pathological articulated peripheral plates (CUWM829 and CUWM980) of a Carettochelyinae turtle (Trionychia, Carettochelyidae) from the Early Miocene of the Moghra Formation (Qattara Depression, Egypt). (**a**) Distal margin, in lateral view, showing the irregular edge and pathological thickening; (**b**) ventral view; (**c**) dorsal view; (**d**) close-up of an anomalous region characterized by exostotic outgrowths and necrotic pitting, in ventral view; (**e**) interpretative schematic drawing, in dorsal view, highlighting key diagnostic features and the extent of bone remodeling; (**f**–**i**) macroscopic transverse sections; (**f**,**g**) corresponding to those of the smaller fragment, showing the fractured margin with the adjacent fragment (**f**) and the opposite margin (**g**); and (**h**,**i**) being sections of the larger fragment, showing the fractured margin with the smaller fragment (**h**), and the opposite margin (**i**). Scale bars represent 2 cm in (**a**–**c**), and 1 cm in (**d**,**f**–**i**). In (**b**), the fragment on the right corresponds to CUWM980, and the one on the left to CUWM829.

**Figure 3:**
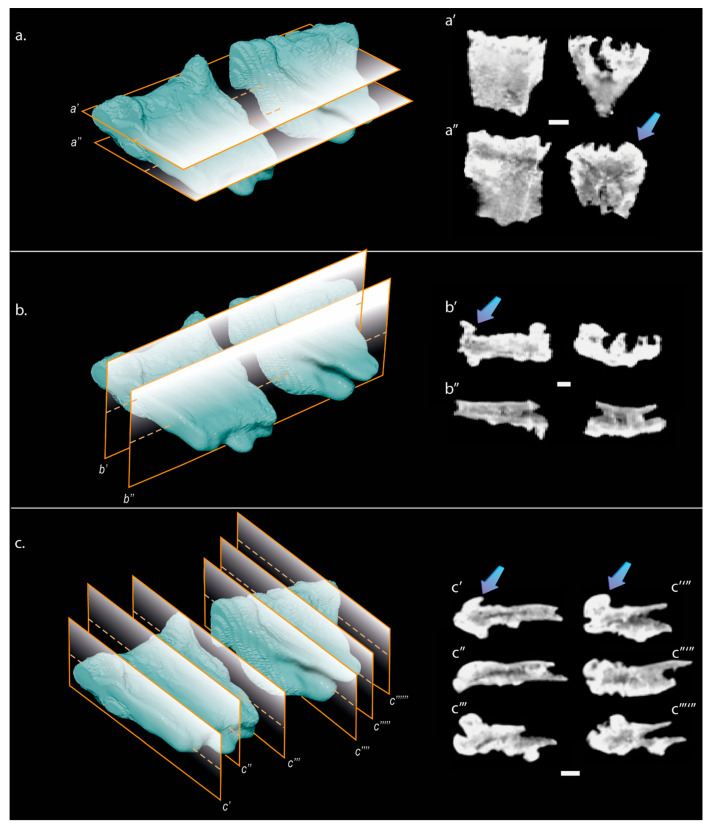
Cross-section slices of two pathological articulated peripheral plates (CUWM829 and CUWM980) of a Carettochelyinae turtle (Trionychia, Carettochelyidae) from the Early Miocene of the Moghra Formation (Qattara Depression, Egypt), obtained by computed tomography (CT). (**a**–**c**), Three-dimensional reconstructions of the articulated peripheral plates showing the position and orientation of the virtual section planes. (**a′**,**a″**), transverse sections along the frontal plane; (**b′**,**b″**), longitudinal sections along the sagittal plane; (**c′**–**c″″″**), multiple serial sections along the transversal plane. Corresponding CT slices (**a′**,**a″**, **b′**,**b″**, **c′**–**c″″″**) are shown to the right of each reconstruction. Arrows indicate areas of increased bone density and pathological bone growth. All scale bars represent 1 cm.

In this context, we describe two anomalous peripheral plates of a carettochelyid turtle from the Moghra Formation (Figure 2). The material was examined through detailed macroscopic observation and computed tomography (CT) imaging (Figure 3) in order to characterize both external and internal structural alterations. These data provide the first evidence of such modifications in an African carettochelyid and contribute to a broader understanding of osseous alteration processes in the Moghra vertebrate assemblage.

## 2. Geologic Setting

The Moghra area is located in the northeastern part of the Qattara Depression (Figure 1a), about 60 km south of El Alamein, in Egypt. The Moghra Formation is the type section for the Lower Miocene deposits in the Egyptian Northwestern Desert. It consists of 400 m of intercalations of siliciclastic sediments (sandstone and shale) [7,17,18,19]. This formation underlies the Middle Miocene Marmarica Formation that crops out on the top of the Qattara escarpment more to the west [5,7,18].

The stratigraphy of the Moghra Formation is defined by nine transgressive-regressive units, each of which is capped by a river-scour surface that severely truncates the underlying regressive half-unit [17]. The transgressive part of each unit is preserved by a deep erosional scour surface and consists of tidal–fluvial sandstones with silicified tree trunks and vertebrate remains [5,17]. Therefore, the Moghra paleoenvironment corresponded to a tide-dominated estuary, displaying series of estuarine units stacked in a net transgressive stratigraphy [5,17,18].

Vertebrate fossils were reported from four stratigraphic horizons of the Moghra Formation (Figure 1b) known as F1, F2, F3, and F4, respectively from the older horizons to the younger ones [5,7,20]. Each horizon is characterized by an erosional lag surface showing mudclasts, coprolites, vertebrate remains, and silicified wood [14].

Strontium isotope (^87^Sr/^86^Sr) age-dating analyses of macrofossil fragments of the Moghra Formation proposed a range from 20.5–17 Ma, corresponding with the Early Miocene (Burdigalian) [18]. The specimens described herein came from the F1 (lower horizon) which was deposited about 19.6–18.2 Ma ago.

## 3. Material and Methods

The described specimens consist of two articulated and almost complete peripheral plates of a turtle shell, separated by a fracture that does not coincide with the suture between them (Figure 2). Based on the characteristic vermiculated ornamentation of their external surface, they are attributable to Carettochelyidae, a clade that has previously been reported in the Egyptian Moghra Formation [16]. The presence of a reduced or absent keratinous covering—a feature definitively absent in members of Carettochelyinae—further supports this identification. The lack of marginal scutes specifically allows for the attribution of the specimen to this clade [21], which has been previously documented within this geological formation. However, given the fragmentary nature of the material and the absence of diagnostic carapacial elements, a more precise taxonomic assignment is not possible. Accordingly, the specimens are referred to as Carettochelyinae indet. They belong to the Moghra collection deposited at the Vertebrate Paleontology Laboratory, Geology Department, Faculty of Science, Cairo University (CUWM). The collection numbers are CUWM829 (the left fragment in Figure 2c) and CUWM980 (the right fragment in Figure 2c).

The material was initially examined from a macroscopic perspective, and a detailed description of the anomalous conditions is provided herein. External morphology and preservation were documented photographically. Photographs for the specimens were taken in natural light using a digital camera (D750 Nikon (Tokyo, Japan); Af-S Nikon 24–120 mm 1:4 G ED VR; Nikon) and lens (Af-S Nikon 70–00 mm 1:28E FL ED VR; Nikon). The anomalies were described in detail, including their morphology, dimensions, and relationship to the surrounding bone tissue. To complement the external examination and further characterize the nature of the observed alterations, the specimens were analyzed using computerized axial tomography (CT). CT scans were performed at the Modern October Scan Center (Egypt) using a Siemens Somatom Sensation 16 clinical scanner (Siemens, Muenchen; Germany), operating at 262 mA and 120.00 kV. This non-destructive technique enabled the evaluation of internal structural features, including cortical thickness, trabecular organization, and potential evidence of bone remodeling associated with the anomalies. The resulting dataset was examined in multiple planes to assess the three-dimensional extent and internal morphology of the lesions. Tomographic data were imported into Avizo 7.1 software (VSG, Germany) for visualization and analysis. This approach facilitated the identification of internal features, such as subsurface density variations and structural discontinuities. The interpretation of the observed alterations was based on systematic comparisons with published medical and veterinary literature on bone pathology, as well as considering the direct observation of extant turtle specimens, particularly representatives of closely related lineages, housed in the Comparative Anatomy and Zoology of Reptiles and Amphibians collections of the Muséum national d’Histoire naturelle (Paris, France). Descriptions were formulated using standardized terminology derived from clinical and osteopathological frameworks. On this basis, a differential assessment was conducted, and a presumptive diagnosis was established through comparison with documented cases in the medical and veterinary literature.

## 4. Systematic Paleontology

Testudines, [22]

Cryptodira, [23]

Trionychia, [24]

Carettochelyidae [25]

Carettochelyinae, [26]

Carettochelyinae indet.

Figure 2 and Figure 3.

Material: Two articulated peripheral plates (CUWM829 and CUWM980).

Locality and horizon: F1 (lower horizon) of Moghra Formation, Egypt, North Africa. Burdigalian (Lower Miocene) [8,18,19].

Description: The studied material consists of two almost complete and articulated pathological plates of a Lower Miocene carettochelyid turtle (Figure 2). That assembly is separated by a fracture. The larger fragment preserves the sutural contact between both adjacent peripherals (Figure 2e). Both plates are characterized by being slightly longer than wide. The outer surface displays an ornamentation pattern consistent with the material previously described for this lineage of freshwater cryptodiran turtles, characterized by prominent thick ridges separated by grooves of subequal width [16].

On the ventral surface (Figure 2b), particularly along the distal margin, the primary ornamentation is interrupted by irregular, anomalous bone outgrowths (exostoses), which exhibit greater development towards the lateral margin. These outgrowths vary in size, ranging from 0.5 cm to 1.0 cm in diameter and reaching heights of up to 1.0 cm. Morphologically, these structures are globular, presenting a smooth and polished cortical surface. Localized regions of necrosis are interspersed among these areas of neoformed bone (Figure 2a), which result in a disorganized and irregular topography. In dorsal view (Figure 2c), the abnormal bone growth extends across nearly the entire surface of the plates, except for the most medial region. This condition is most pronounced at the distal margin, where the accumulation of anomalous bone creates a distinctly thickened ridge. In transverse section (Figure 2f–i), the contact between the original cortical bone and the neoformed bone is abrupt and clearly defined. The anomalous bone exhibits a significantly higher density compared to the healthy bone tissue, indicating an intensive ossification process in the affected areas.

CT data confirms that these anomalous outgrowths involve profound internal remodeling of the peripheral plates (Figure 3). Orthoslices reveal significantly increased radiodensity within the exostotic masses compared to healthy bone, identifying them as sclerotic reactive tissue. These hyperostotic regions are interspersed with localized low-attenuation voids, which correlate with the necrotic pits observed macroscopically.

## 5. Discussion

### 5.1. Pathological Characterization and Diagnosis

The interpretation of pathological shell alterations in turtles is relatively complex due to the strong morphological overlap between biological, pathological, and taphonomic processes [27,28]. Previous studies on fossil turtles have documented a broad spectrum of shell modifications, ranging from discrete pits and grooves to perforations [29], marginal damage [30], and irregular areas of surface loss [31], but these features are not always accompanied by evidence of host response. As a result, similar external morphologies have been assigned to different causes in the literature, including predatory interactions [32], invertebrate activity [33], microbial degradation [27], environmental abrasion [34], and disease-related shell alteration [35]. This diagnostic ambiguity is particularly relevant for aquatic and semi-aquatic turtles, whose shells are continuously exposed to mechanical wear, epibionts, parasites, and waterborne microorganisms, all of which may affect the external surface without necessarily producing deep osseous remodeling. Studies of extant turtles provide useful clinical analogues for recognizing shell damage and repair, but many of them emphasize epidemiology, husbandry, or gross clinical appearance rather than the osteological criteria needed for paleopathological diagnosis [36,37,38]. Conversely, reports of disease-related lesions in fossil reptiles show that internal structural change, organized bone deposition, and remodeling patterns are essential for distinguishing true premortem pathologies from superficial modifications or post-mortem alterations [27]. In this sense, the comparative literature highlights that turtle shell lesions should not be interpreted on the basis of external morphology alone, but require integration of surface anatomy, internal architecture, and the broader paleoenvironmental context.

Thus, the distinction between pathological bone remodeling and post-mortem taphonomic alteration in the studied specimens is based primarily on the presence and organization of reactive bone tissue. Bone remodeling processes, including cortical thickening, exostotic formation, and evidence of internal structural reorganization, are considered strongly indicative of a biological response occurring during the life of the organism. In contrast, post-mortem alterations typically lack organized microstructural modification of the original bone tissue and do not show evidence of coordinated bone deposition or resorption. Accordingly, surface and internal modifications associated with clearly developed remodeling patterns are interpreted here as the result of pathological processes affecting the living animal, whereas features lacking such structural bone response are treated cautiously and not attributed to premortem conditions without additional supporting evidence. The pathological features observed in the carettochelyid peripheral plates studied here are consistent with a complex, polyphasic infectious osteopathy, most parsimoniously interpreted as an advanced stage of osteomyelitis. The characterization as complex reflects the concurrent manifestation of opposing physiological processes, namely bone resorption (osteolysis) and reactive bone formation (osteogenesis) [39,40,41]. In the studied specimen, this duality is expressed through nodular exostoses that largely obscure the primary carettochelyid ornamentation, in association with focal necrotic depressions (Figure 2d).

The heterogeneous surface morphology supports a polyphasic progression, indicating repeated or prolonged episodes of pathological activity [40]. The coexistence of relatively smooth areas with irregular, rugose textures is consistent with alternating phases of partial stabilization and renewed inflammatory activity [41,42].

Computed tomography data further supports a substantial internal reorganization of the bone. The increased radiodensity within the exostotic regions is indicative of a marked sclerotic response [43], with the affected bone exhibiting higher density relative to the unaffected cortical tissue (Figure 3). This process involves extensive infilling of internal trabecular spaces, reducing normal bone porosity [40].

According to Jacobson (2007), such pronounced mineralization is consistent with a predominantly sclerotic (blastic-like) response, as described for chronic osteomyelitis in extant reptiles [40]. The disorganized internal architecture, characterized by dense mineralized tissue interspersed with lower-attenuation voids, reflects the coexistence of osteolytic and osteosclerotic processes [44,45,46,47].

Alternative interpretations, including neoplastic processes or isolated traumatic periostitis, appear less consistent with the observed morphology and internal structure. Neoplastic conditions in turtles, although documented in both extant and extinct taxa [45,48,49,50,51], typically are represented as discrete, autonomous masses with more defined margins than the lesions described here [45]. In contrast, the combination of extensive reactive sclerosis, deep cortical remodeling, and localized necrotic pitting identified in CUWM829 and CUWM980 reflects a generalized inflammatory response rather than an isolated proliferative growth [40,45,52]. Furthermore, the polyphasic nature of the lesions, characterized by alternating cycles of bone resorption and apposition, is highly diagnostic of chronic infectious osteomyelitis [40]. This differs significantly from the more uniform healing callus typically associated with a single traumatic event or a localized periostitis [53,54,55], which generally lacks the systemic internal infilling observed in the field obtained here by the use of the CT scan.

Although the precise duration of the pathological condition cannot be established, the degree of cortical remodeling and the extent of neoformed bone indicate that the individual survived long enough for the development of a substantial reactive bone deposition. However, the incomplete integration and remodeling of the affected areas suggest that the pathological process was ongoing at or near the time of death.

Nevertheless, it is important to recognize that the present diagnosis remains presumptive, as is inherent to most paleopathological interpretations based exclusively on fossil material. Although the combined macroscopic and computed tomographic evidence provides a robust basis for interpretation, the absence of histological thin sections constitutes a relevant methodological limitation. Histological analysis would have allowed the assessment of bone microarchitecture, including tissue organization and vascular patterns, as well as the identification of remodeling features such as secondary osteons and resorption structures. In this context, such data could have significantly strengthened the interpretation of the lesions; however, their absence does not preclude a confident differential diagnosis based on the available evidence. Accordingly, the pathological condition described herein is most appropriately regarded as consistent with an osteomyelitic process, within the methodological constraints of the study.

### 5.2. Potential Etiology

In aquatic turtles, the structural integrity of the shell constitutes the primary physical barrier against opportunistic environmental pathogens. Disruption of this cornified layer provides a potential portal of entry for opportunistic microorganisms, which may subsequently colonize the vascularized subdermal tissues [40]. Given the aquatic ecology of carettochelyids, one plausible pathway for the development of the infectious process observed in the studied specimen involves localized mechanical disruption of the shell surface.

The fossil material does not preserve direct evidence that allows the identification of a specific initiating factor for such disruption. Although the absence of diagnostic traces of vertebrate predation (e.g., puncture marks, striations, or serrated damage) [56] rules out clear evidence of predatory interaction, it does not preclude other mechanical or environmental causes. In high-energy depositional settings, non-lethal impacts or sustained abrasive contact may produce focal microdamage in the shell, although such processes cannot be directly demonstrated in the present material. As documented in extant taxa [57,58], similar superficial disruptions may facilitate the infiltration of opportunistic aquatic bacteria (e.g., *Aeromonas*), potentially leading to secondary infection of the underlying bone tissues [40]. Alternatively, the observed condition may be broadly comparable, in terms of general pathological outcome, to the progression of septicemic cutaneous ulcerative disease (SCUD) or advanced shell rot in extant turtles [40,59,60,61]. These conditions typically originate as localized necrosis of the shell and, if unresolved, may extend into the underlying bone [61,62]. Once the infection reaches the vascularized cancellous bone, it can trigger extensive internal remodeling and marginal thickening, consistent with the structural alterations identified through the CT analysis conducted herein [40,44]. In this context, modern pathological analogues are used strictly as comparative models to illustrate potential pathways of infection, rather than as direct diagnostic equivalents for the fossil specimen. The concentration of lesions toward the distal margins of the plates is compatible with an environmental entry point, where the shell may be more susceptible to both mechanical damage and microbial colonization.

### 5.3. Paleoecological Context

The high frequency of shell modifications documented in the Moghra Formation reflects a complex ecological dynamic where different turtle species were subject to distinct biological pressures [31]. Ref. [15] described a diverse assemblage of ichnotaxa affecting both terrestrial (testudinids) and aquatic (podocnemidids and trionychids) taxa of Testudines [31]. The primary biological threats recognized for these authors in the aquatic turtles of the Moghra ecosystem included ectoparasitic borings and microbial shell rot, which are significantly more prevalent in aquatic taxa.

The advanced osteomyelitis observed in the carettochelyid studied here represents a severe progression of these environmental interactions. While ref. [15] noted that mesoparasite borings and microbial pitting were common among the aquatic turtles of the Moghra Formation, the systemic sclerotic remodeling evidenced in this specimen suggests that these focal lesions could escalate into life-threatening infections under significant pathogenic pressure. In a tide-dominated estuary like that where the Moghra Formation was deposited, the constant presence of leeches and trematodes (as suggested by the *Karethraichnus lakkos* and *Thatchtelithichnus* traces; see [15] and opportunistic microbial pathogens represents an elevated biological pressure on the structural integrity of the Testudines shell, particularly for aquatic taxa.

### 5.4. Paleobiological Implications

Paleopathological records in fossil carettochelyids remain exceptionally scarce, limiting current understanding of disease processes within this lineage [63]. The specimen described herein therefore represents an important addition to the paleobiological record of this clade and provides rare evidence of chronic infectious disease in a trionychian turtle from the African Miocene.

The degree of reactive bone formation and cortical remodeling identified in CUWM829 and CUWM980 reflects a potentially prolonged survival period despite a chronic infection [44]. This indicates a robust physiological resilience, where the metabolic rate of the animal allowed for a long-term immune response [57,61,62]. The extensive sclerosis and internal stabilization observed via the field obtained by the use of the CT scan suggest that carettochelyids, like many extant turtles, could maintain functional integrity of the shell even under significant pathological stress [62].

Furthermore, the anatomical characteristics of the Carettochelyinae (i.e., particularly the absence of keratinous scutes and the presence of a naturally rugose outer shell ornamentation) [64] likely influenced their interaction with the environment [65,66]. In high-energy estuarine settings, the lack of a keratinous barrier may have increased the susceptibility of the dermal bone to direct microbial colonization following minor abrasions.

## 6. Conclusions

This study provides the first detailed description of pathological bone modifications in African carettochelyid, based on two articulated peripheral plates recovered from the F1 horizon of the Moghra Formation (Early Miocene, 19.6–18.2 Ma). The integration of macroscopic observation and the analysis of data acquired via computed tomography (CT) has allowed for a comprehensive characterization of both the external morphology and the internal structure of the plates. The identified anomalies include globular exostoses that have completely replaced the primary ornamentation, alongside necrotic areas and internal sclerosis evidenced by the filling of internal bone pores. These features are consistent with an advanced osteomyelitis. The simultaneous presence of bone resorption and reactive formation indicates a sustained host–pathogen interaction, but the individual survived long enough to allow for significant osteogenic remodeling.

The observed condition likely resulted from the structural compromise of the shell plates caused by mechanical trauma or erosion, which provided a portal of entry for opportunistic aquatic pathogens. This type of infectious onset is common in aquatic taxa, where the constant exposure to the surrounding medium facilitates the infiltration of microorganisms once the defenses of the shell are compromised. The subsequent formation of dense new bone reflects an active physiological response aimed at isolating the infection and preserving the structural integrity of the peripheral margins.

From a paleoecological perspective, these findings complement the ichnological data previously documented on the shells of Testudines within the Moghra Formation [31]. While previous traces illustrate parasitic and microbial prevalence in the turtle and tortoise shells from the Moghra Formation, this specimen demonstrates how such interactions could escalate into extensive bone disease in fully aquatic taxa.

## Data Availability

No datasets were generated or analysed during the current study.
